# An efficient machine learning-based approach for screening individuals at risk of hereditary haemochromatosis

**DOI:** 10.1038/s41598-020-77367-6

**Published:** 2020-11-26

**Authors:** Patricia Martins Conde, Thomas Sauter, Thanh-Phuong Nguyen

**Affiliations:** 1 Megeno S.A, Esch-sur-Alzette, Luxembourg; 2 University of Luxembourg, Esch-sur-Alzette, Luxembourg

**Keywords:** Risk factors, Machine learning, Predictive medicine

## Abstract

Hereditary haemochromatosis (HH) is an autosomal recessive disease, where *HFE* C282Y homozygosity accounts for 80–85% of clinical cases among the Caucasian population. HH is characterised by the accumulation of iron, which, if untreated, can lead to the development of liver cirrhosis and liver cancer. Since iron overload is preventable and treatable if diagnosed early, high-risk individuals can be identified through effective screening employing artificial intelligence-based approaches. However, such tools expose novel challenges associated with the handling and integration of large heterogeneous datasets. We have developed an efficient computational model to screen individuals for HH using the family study data of the Hemochromatosis and Iron Overload Screening (HEIRS) cohort. This dataset, consisting of 254 cases and 701 controls, contains variables extracted from questionnaires and laboratory blood tests. The final model was trained on an extreme gradient boosting classifier using the most relevant risk factors: *HFE* C282Y homozygosity, age, mean corpuscular volume, iron level, serum ferritin level, transferrin saturation, and unsaturated iron-binding capacity. Hyperparameter optimisation was carried out with multiple runs, resulting in 0.94 ± 0.02 area under the receiving operating characteristic curve (AUCROC) for tenfold stratified cross-validation, demonstrating its outperformance when compared to the iron overload screening (IRON) tool.

## Introduction

Iron overload is characterised by the accumulation of iron in the body, and the primary cause for this condition is hereditary haemochromatosis (HH). HH is an autosomal recessive genetic disease associated with the C282Y homozygosity in *HFE* gene, accounting for 80–85% of HH cases in the Caucasian population^1^. This condition is characterized by increased iron absorption rates, and leads to the accumulation of iron (iron overload), mainly in the liver, heart and endocrine glands^2^. If left untreated, the iron overload leads to the development of liver cirrhosis and liver cancer, decreasing life expectancy^3^. These complications can be prevented by phlebotomy therapy if diagnosis is made before any organ damage occurs. Thus, it is of utmost importance to identify individuals at risk of iron overload in order to maximise on early prevention and/or early intervention measures^4^, reduce treatment costs and improve HH individuals’ life expectancy.

Commonly, high risk individuals can be identified based on their family health history, clinical biomarkers, and monogenic risk or on polygenic risk scores. Even though different sources of data can be used to estimate disease-specific risk, most common risk assessment models rely only on one specific type of data. For example, family health history is widely integrated in the risk assessment of common chronic diseases, and it has been shown that an individual’s risk is proportional to the number of relatives affected by the disease^5^. While for some diseases, such as hereditary breast and ovarian cancer, the risk can be assessed solely on family health history, for others, such as HH, risk assessment tools also integrate clinical and demographic information^5^.

Multiple *HFE*-associated HH cohorts, such as the Hemochromatosis and Iron Overload Screening (HEIRS)^3^, HealthIron^6^, Southern French registry^7^, have been investigated to identify the genetic and environmental modifiers of iron-overload phenotypes, as well as the HH clinical prevalence and genotype penetrance. Among those, the largest available cohort is the HEIRS cohort, where more than 100,000 individuals were enrolled in the United States and in Canada. Multiple studies have extensively investigated the HEIRS cohort, and key insights were reviewed in McLaren and Gordeuk (2009). In addition, data from the HEIRS subpopulations have influenced the development of a model enabling the identification of *HFE* C282Y homozygous in Caucasians^8^, and has been investigated for associations between clinical variables and hyperferritinemia^9^ in African Americans. However, only a small number of studies have explored the family study dataset, demonstrating the heritability of serum iron markers^10^, and the potential contribution of quantitative trait loci to variation in these measures^11^.

Not only *HFE*-associated HH cohorts have been explored, but also subpopulations of general population cohorts have been analysed, such as the UKBiobank, and the National Health and Nutrition Examination Survey (NHANES) cohorts. Studies on these subpopulations have substantiated a comparison of prevalence and morbidity between individuals with and without *HFE* C282Y genetic variants^12^, leveraging the development of iron overload screening tools that could be used in primary care^13–15^.

To the best of our knowledge, only two iron-overload/HH screening models have been proposed. The iron overload screening tool (IRON)^13^, which is based on traditional statistical methods on self-reported and non-laboratory data, obtained an area under the receiving operating characteristic curve (AUCROC) of 68.5%. Other studies have investigated how phenotype data (clinical symptoms) alone, extracted from electronic health records^14^, or clinical symptoms integrated with laboratory data perform in HH risk prediction^15^. They demonstrated that the phenotype risk score (PheRS) based only on clinical symptoms achieved an AUCROC between 65 and 69%, similar to the IRON score. On the other hand, AUCROC was significantly increased when phenotypic and laboratory data were integrated (AUCROC between 84 and 85%). These results demonstrate the great potential of integrating different types of biomedical data into computational screening tools to create a holistic view of both the healthy and unhealthy individual^16^.

Recent advances in big data analytics and the availability of large biomedical datasets actualise advancements within personalised medicine, substantiating a tailored diagnostic, therapeutic and preventive approach to every individual. Personalised medicine benefits from a targeted phenotypic surveillance of diseases for which a genetic predisposition exists, and provides a unique opportunity to identify individuals at different risk levels for specific diseases. However, the integration of large biomedical data brings upon new computational challenges such as the curse of dimensionality, data heterogeneity, missing data, class imbalance and scalability^17^. These challenges can be overcome by utilising specialised computational methods, such as machine learning (ML), to efficiently integrate and process massive amounts of heterogeneous information^17^. Furthermore, the high dimensionality of the available information renders it impossible for a human being to decipher the most significant clinical information^16^. To address this, ML is being employed to convert large complex heterogeneous datasets into simple, interpretable and actionable information. Such an approach is extremely useful in isolating dependencies between variables, which is not possible when using univariate methods, thereby translating into more accurate predictive models^18^. Despite the advances in data integration and data analytics, the ultimate goal of automatic integration of heterogeneous data remains unmet^18^.

Current approaches employed to assess HH risk or *HFE* C282Y homozygosity are very simplistic and utilise methods such as Cox or logistic regression analysis^8,13^. Even though they have shown promising results, more robust and accurate risk prediction models are necessary. Torkamani et al*.* and Ginsburg et al*.* have suggested that the usage of robust methods, such as ML algorithms, together with the integration of a panoply of personal information (family health history, genetic variants, clinical and lifestyle data), are the future of personalised medicine^19^.

In this study we aim to develop a new risk stratification model for HH based on the family study data from the HEIRS cohort, and will investigate the following hypotheses: (1) the integration of family health history, demographic, clinical and genomic data should improve the accuracy of risk assessment models, (2) ML models enable the integration of large scale heterogeneous data and the extraction of valuable knowledge, thereby improving the performance of predictive models. To address these hypotheses, a ML-based pipeline is proposed, enabling the identification of the most clinically relevant risk factors and the selection of the best risk model.

## Methods

**Figure 1:**
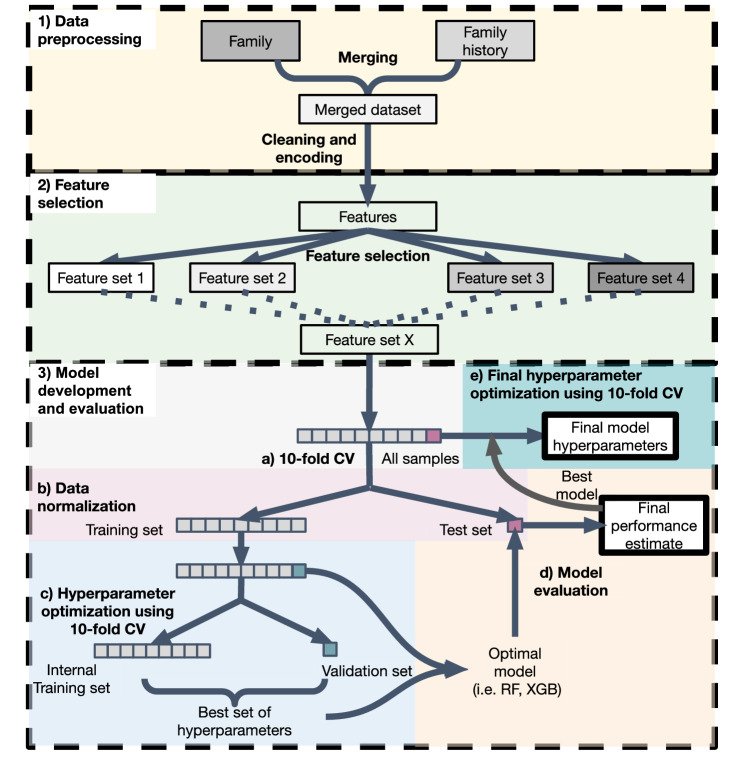
Workflow for the construction of a HH risk model based on machine learning. This workflow consists of three steps: (1) data preprocessing, (2) feature selection, and (3) model development and evaluation. In the first step, the family and family history datasets from the family study were merged. The family data set contains data from different sources, i.e., demographics (age, gender, and ethnicity), blood markers, and personal medical history. The data was cleaned and categorical variables with more than two classes were encoded using an one-hot-encoding approach. In the second step, feature selection based on six different methods (statistical and machine learning-based) was performed and eight different sets of risk factors were manually selected. In the last step, each of the selected risk factor sets were evaluated using different machine learning algorithms. First, the data was split into training and testing sets using tenfold stratified cross-validation (CV). The hyperparameters of each ML algorithm were tuned using GridSearch and tenfold stratified CV, and optimized for F1 score. After hyperparameter optimization, the optimal model was trained and evaluated on an unseen test set. This step was repeated 10 times. After final performance estimate, the best model including the best feature set were selected, and hyperparameter optimization was run on the whole dataset using GridSearch and tenfold stratified CV.

This section describes our proposed ML-based method combining on multiple family health history, demographic, clinical and genomic data. Figure 1 demonstrates the overall workflow.

### Data description

The family study from the HEIRS^3^ cohort was employed to develop and assess the performance of a HH risk prediction model. In the HEIRS cohort, more than 100,000 adults (> 25 years old) were enrolled over a period of 2 years at four centres in the USA, and one in Canada, in order to investigate the prevalence, the determinants and the potential multi-level effects of HH in a multi-ethnic population sample^9^. To identify individuals suffering from HH, in the HEIRS study the following steps were taken: (1) in the initial screening, individuals were screened for *HFE* C282Y and H63D mutations, and serum ferritin and transferrin saturation levels were measured. Based on these measures, HH cases and controls were identified. (2) HH cases and frequency matched 1:1 controls were invited for a comprehensive clinical examination (CCE). Variables used for the frequency matching included age, gender, ethnicity, field centre, and date of the initial screening. (3) Eligible cases and their relatives were enrolled for the family study. (4) All CCE individuals except controls were followed up after 1 year. All individuals invited to the CCE filled questionnaires on their medical history, medications, supplements, family pedigree, family health history, and food frequency, with the exception of the control group, which did not complete any family related questions.

In this work, we were interested in predicting HH in a familial context. Therefore, we investigated the family study from the HEIRS^3^ cohort, which included data from 997 individuals, original cases from the HEIRS cohort that met the family study inclusion criteria and their family members. We used anonymised data that were already collected and published and therefore no patients were involved in setting the research question or the outcome measures. All of the individuals considered had completed a personal and a family health history questionnaire. Additionally, genotype (i.e., mutations in *HFE* gene), laboratory blood values (i.e., transferrin saturation, serum ferritin concentration) and demographic information (i.e., age, gender, and ethnicity) were also available. In this study, only the data available in the family and family history datasets were considered. After merging the data from these two datasets, the cohort included a total number of 955 individuals (254 cases and 701 controls), leading to the imbalance between cases and control. Key descriptive statistics of the merged dataset are shown in Table 1.

**Table 1:** Characteristics of the family study participants (n = 955).

	Controls (%)	Cases (%)
**Gender**		
Male	30.58	12.57
Female	42.83	14.03
**Age**		
< 20	1.99	0.21
20–29	10.89	1.88
30–39	14.97	2.72
40–49	17.17	7.02
50–59	12.15	6.07
≥ 60	16.23	8.69
**Race/ethnic group**		
Caucasian	56.75	19.27
Asian/Pacific Islander	6.81	4.19
Hispanic	6.28	1.68
African American	2.2	0.84
American Indian, Multiple, Unknown	1.36	0.63
**Genotype**		
Healthy	20.94	4.92
*HFE* C282Y homozygosity	6.7	15.92

The healthy genotype represents individuals with no C282Y or H63D mutation in *HFE* gene.

### Data preprocessing

Information about an individual’s family health history is available in the family history dataset. Each individual completed a questionnaire reporting the relationship, gender, age at diagnosis and the medical health history for seven specific diseases for blood and non-blood relatives i.e., parents, siblings, spouse, etc. In this study, we were interested in predicting HH cases in a familial context. Thus, we derived two new variables from this dataset for each reported disease (haemochromatosis, diabetes, alcoholism, liver cancer, liver cirrhosis, arthritis and heart disease). The first variable represents the total number of blood relatives affected by e.g., haemochromatosis (NumRel_hemo). Blood relatives included parents, siblings, children, grandparents, grandchildren, cousins, uncles, aunts, nephews and nieces. The second derived variable describes the family health history, where a positive, and a negative family health history were encoded as 1, and 0, respectively. A positive family health history for e.g., haemochromatosis (Rel_hemo) was defined by at least one blood relative being diagnosed with haemochromatosis. Finally, the family dataset and the family health history were integrated by mapping the subject ids.

Features containing more than 90% of missing values were removed from the final set. The remaining missing values were imputed in the following manner: firstly, the majority class of a feature was imputed if the missing value belonged to a category, such as gender. Otherwise, the median of a feature was used if the missing value was numerical, such as age.

Categorical variables encoding more than two classes, i.e., genotype, were encoded using one-hot-encoding. For the ones encoding only two classes, i.e., gender or positive history of disease, the classes were converted to 0 or 1. For gender, female individuals were encoded as 1 and males as 0. For the variable rhMen (being at menopause), individuals at menopause were encoded as 1. In addition, all males were considered to be always at menopause.

The data was normalized using the default StandardScaler function from *sklearn*. First, the training data was centred and scaled prior to the model training, and then the testing data was normalized to the training one. This step was performed after splitting the data to avoid any bias during the evaluation of the models.

In this study, cases were defined as individuals with (1) a provisional classification of primary iron overload, as established in the HEIRS cohort, such as individuals with elevations of both serum ferritin concentration (> 200 ng/ml women; > 300 ng/ml men) and transferrin saturation (> 45% women; > 50% men) and no evidence of inflammation, hepatocellular dysfunction or secondary iron overload, or (2) individuals that self-reported a medical history of haemochromatosis. Based on these two criteria, a total of 254 cases among 955 individuals were identified.

### Feature selection

Feature selection was performed in order to reduce the dimensionality of the dataset, and identify the most relevant risk factors for HH screening. Furthermore, this step also helps to increase the efficiency of ML models, as irrelevant data are removed.

Multiple feature ranking (FR) methods available on *sklearn* and* statsmodels* libraries were selected in order to assess how the ranking varied among them. The first group of methods, which are independent of a threshold, included (A) Wilcoxon signed-rank test followed by Bonferroni correction, (B) extreme gradient boosting and (C) random forests employing both tenfold stratified cross-validation (CV) and recursive feature elimination (RFE), and optimized for F1 score. The second group of methods, which are dependent on a threshold, encompassed (D) mutual information, (E) extreme gradient boosting, and (F) random forests. The full feature ranking results are shown in Table 2.

**Table 2:** List of top risk factors obtained after feature ranking. Multiple methods were used to extract the most relevant variables.

A	B	C	D	E	F
uibc^21,22^	uibc^21,22^	uibc^21,22^	ts^23^	uibc^21,22^	uibc^21,22^
ts^23^	ts^23^	ts^23^	uibc^21,22^	ts^23^	sf^24^
fer^24^	C282Y/C282Y^24^	sf^24^	C282Y/C282Y^24^	C282Y/C282Y^24^	C282Y/C282Y^24^
sf^24^	sf^24^	C282Y/C282Y^24^	sf^24^	sf^24^	ts^23^
C282Y/C282Y^24^	gender^25^	fer^24^	fer^24^	gender^25^	fer^24^
tibc^26^	ast^27^	alt^27^	C282Y/ + ^28^	ast^27^	tibc^26^
mch^29^	fer^24^	age^30^	plt	fer^24^	mch^29^
mcv^29^	age^30^	plt	mcv^29^	Caucasian^24^	mcv^29^
C282Y/ + ^28^	alt^27^	tibc^26^	tibc^26^	age^30^	ast^27^
rdw	mcv^29^		age^30^	aneut	C282Y/ + ^28^
age^30^	Asian/PacificIslander^21^		mch^29^	mcv^29^	rdw
hgb	NumRel_hemo^25^		wmono	alt^27^	alt^27^
hct	rbc		Rel_hemo^25^	rbc	age^30^
rhMen^25^			mhArth^26^	hgb	ggt
ggt			alt^27^	Perso_arthrit^26^	plt

(A) Wilcoxon signed-rank test followed by Bonferroni correction. Only features with an adjusted p-value ≤ 0.05 are shown. (B) Extreme gradient boosting and (C) random forests employing both tenfold stratified CV and RFE, and optimized for F1 score. In columns D to F, only the top 15 features are shown, and these were obtained by employing (D) mutual information, (E) extreme gradient boosting, and (F) random forests. All risk factors are sorted by decreasing order of significance.

*alt* alanine aminotransferase serum activity, *aneut* absolute number of neutrophils, *Asian/PacificIslander* Asian or Pacific Islander ethnicity, *ast* aspartate aminotransferase serum activity, *C282Y/ +* *HFE* C282Y heterozygosity, *C282Y/C282Y* *HFE* C282Y homozygosity, *Caucasian* Caucasian ethnicity, *fer* serum iron concentration, *ggt* gamma glutamyl transferase serum activity, *hct* haematocrit, *hgb* haemoglobin concentration, *mch* mean corpuscular haemoglobin/RBC, *mcv* mean corpuscular volume, *mhArth* positive medical history of arthritis, *NumRel_hemo* number of relatives affected by haemochromatosis, *Perso_arthrit* personal history of arthritis, *plt* platelet count, *rbc* red blood cell count, *rdw* red blood cell distribution width, *Rel_hemo* positive family history of haemochromatosis, *rhMen* at menopause, *sf* serum ferritin concentration, *tibc* total iron binding capacity, *ts* transferrin saturation, *uibc* unsaturated iron binding capacity, *wmono* % monocytes in whole blood cell count.

After feature ranking, we tested different combinations of risk factors for model building. Each feature ranking method returned a set of risk factors. The produced feature sets were overlapping, but were not exactly the same. In addition, the feature ranking results of methods D, E and F were not used for further analysis, as no CV was employed.

1. Set A includes all the statistically significant risk factors obtained by Wilcoxon signed-rank test followed by Bonferroni correction (*adjusted p-value* ≤ 0.05).
2. Set B includes all the risk factors obtained using RFE with tenfold stratified CV extreme gradient boosting and optimized for F1 score.
3. Set C includes all the risk factors obtained using RFE with tenfold stratified CV random forests and optimized for F1 score.
4. Set A&B includes the common risk factors between sets A and B.
5. Set A&C includes the common risk factors between sets A and C.
6. Set B&C includes the common risk factors between sets B and C.
7. Set A&B&C includes all the common risk factors between set A, B and set C.
8. Set ALL includes the complete dataset (a total of 122 features).

Each of these feature sets were fed independently to the pipeline shown in Fig. 1, Step 3.

### Model development

After selecting multiple sets of risk factors that were relevant for HH, we evaluated which of these sets performed better in the classification of HH. To achieve this, an integrative pipeline (Fig. 1) consisting of several steps was developed to test and evaluate the performance of multiple machine learning algorithms, such as logistic regression (LR), decision trees (DT), random forests (RF), extreme gradient boosting (XGB), multilayer perceptron (MLP), support vector machine (SVM) and k-nearest neighbours (KNN) on each set of the risk factors.

- For each set of risk factors, the input dataset was split into a training and a testing set. The same training and testing sets were used to test each algorithm and each feature set.
- The training set was normalized, and then the test set was normalized to the training set.
- The hyperparameters of each ML algorithm were optimised for F1 score using GridSearch and tenfold CV on the training set.
- Each ML algorithm was trained with the best found hyperparameters and evaluated on an unseen test set. Different metrics were calculated in order to evaluate the validity of the models:
  1 $$Accuracy=\frac{TP+TN}{TP+ TN + FP + FN}$$
  2 $$Sensitivity=\frac{TP}{TP+ FN}$$
  3 $$Specificity=\frac{TN}{TN + FP}$$
  4 $$Precision=\frac{TP}{TP + FP}$$
  5 $$F1 Score= 2\times \frac{Precision \times Sensitivity}{Precision + Sensitivity}$$
  where TP (true positives) represents the number of sick individuals correctly identified as sick, TN (true negatives) corresponds to the number of healthy individuals correctly identified as healthy, FP (false positives) represents the number of healthy individuals incorrectly identified as sick, and FN (false negatives) describes the number of sick individuals incorrectly identified as healthy.
  6 $${\text{Area under the receiving operating characteristic curve }}(\mathrm{AUCROC})$$
  7 $${\text{Area under the precision}}-{\text{recall curve}}(\mathrm{AUPRC })$$
  This step was repeated using tenfold CV to allow the estimation of the model generalization.
- The best performing model was selected and tenfold CV GridSearch hyperparameter tuning optimising for F1 score was performed on the full dataset.

This pipeline was implemented and run on Python 3.6.4 (https://www.python.org/) for each of the input datasets described in the Feature selection subsection.

### Model benchmark

Previously, an iron overload screening tool (IRON score^13^), based on an individual’s medical history and symptoms, was proposed for iron overload screening in primary care. This model was trained and validated on the NHANES 1999–2002, and NHANES III datasets, respectively. These datasets are part of a national representative cohort of the US population, and include physical exams, laboratory tests and interviews. Candidate risk factors, known to be associated with haemochromatosis and iron overload, were manually pre-selected for feature ranking. The final risk factor selection was performed using backward elimination followed by logistic regression for p-value assessment. The final IRON score model was implemented in several steps. (1) The final list of risk factors (age, gender, medical history of liver disease, osteoporosis and thyroid disease) was trained with logistic regression. (2) The odds ratios (OR) were extracted for each risk factor. (3) A score was assigned for each range of OR i.e., OR between 1.3 and 1.49 received a score of 1. (4) For each individual, the final IRON score was calculated by summing up the scores of each risk factor. To the best of our knowledge, this is the only readily available model published for HH. Thus, the IRON score model was utilised with two novel strategies in order to benchmark our final HH risk model. The first strategy directly applied the IRON score to the HEIRS family study cohort. Firstly, the five risk factors used in the IRON score were identified (age, gender, medical history of liver disease, osteoporosis and thyroid disease). Secondly, age was discretized, and each independent risk factor received a score, as previously defined^13^. In the second strategy, the risk factors selected for the IRON score were used as input variables for the machine learning pipeline described in the previous subsection.

Other risk score models based only on clinical symptoms, or integrating these with laboratory data have recently been proposed^14,15^. However, these models are not available and reproducible, including the script, model parameters, HPO terms, laboratory variables and ICD-codes.

### Ethical approval

Ethics Review Panel of the University of Luxembourg approved the project "A computational approach to hemochromatosis and iron overload surveillance (CARES)" (ERP 19–034), including HEIRS data analysis.

## Results

In this work, we have analysed the family study dataset, which is part of the HEIRS cohort, to identify the most important risk factors for HH and to propose a new risk screening model for this disease.

### Descriptive analysis of the family study participants

The characteristics of the family study cohort are described in Table 1. In this cohort, around 73% of the individuals were between 20 and 60 years old. Additionally, an increase in the percentage of HH cases was visible in individuals older than 40 years old. Around 76% of the individuals were of Caucasian background, and of those 19% were cases. Asian/Pacific Islanders and Hispanics had a similar number of control cases, however more HH cases were present in individuals of Asian/Pacific Islander background.

Around 26% of all individuals did not have any C282Y nor H63D mutation in the *HFE* gene, and among these around 20% had HH. On the other hand, around 23% of all individuals were *HFE* C282Y homozygous, and around 70% of them had HH.

More than 80% of *HFE* C282Y homozygotes will suffer from iron overload. However, the determination of *HFE* genotype is only recommended if serum ferritin concentration and transferrin saturation values^20^ are above gender specific reference ranges. Additionally, it has been reported that because of blood mensal losses, non-menopause females show lower serum ferritin and transferrin saturation levels when compared to males. Furthermore, previous data has shown that unsaturated iron binding capacity (uibc) performs equally well as transferrin saturation in the identification of *HFE* C282Y homozygotes^21^. Thus, we were interested in assessing how these variables varied among the different *HFE* genotypes and gender in the family study.

**Figure 2:**
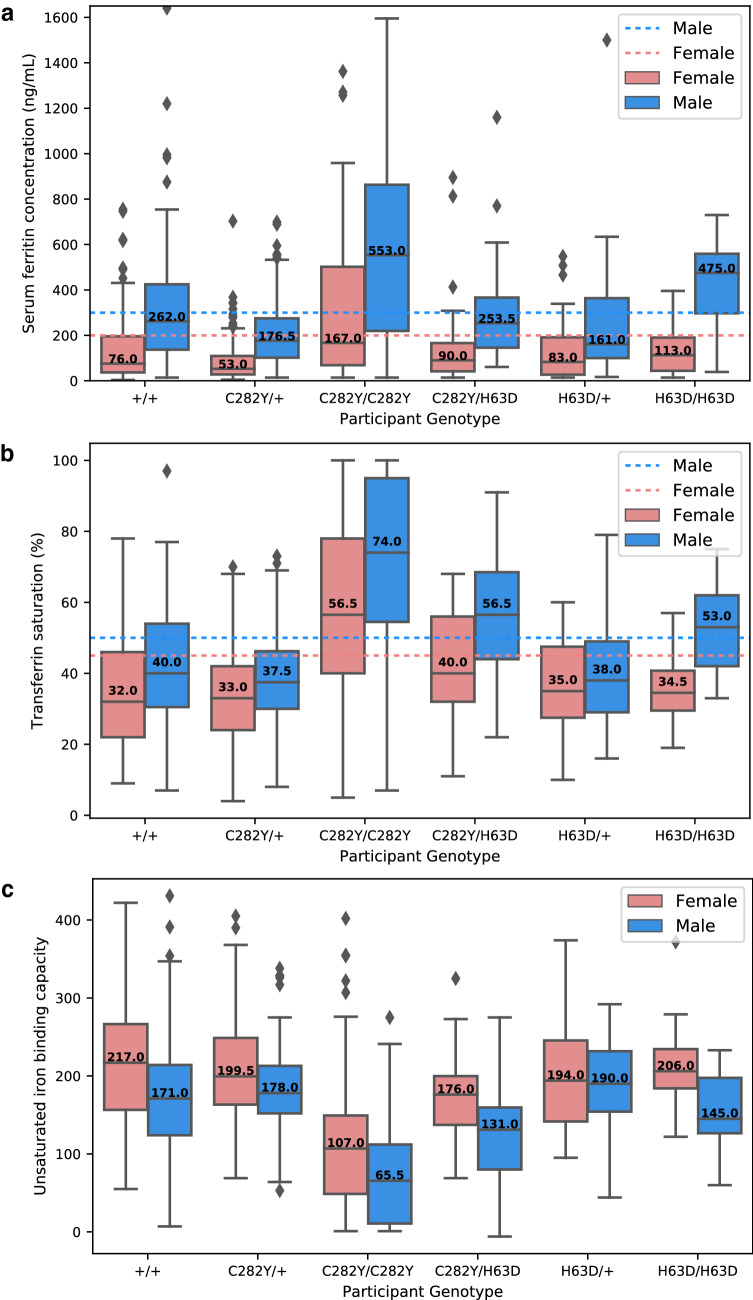
Distribution of (**a**) serum ferritin concentration, (**b**) transferrin saturation and (**c**) unsaturated iron binding capacity among the different *HFE* genotypes. The red and the blue dashed line represent the reference range for female and male individuals, respectively. The reference ranges for females are 200 ng/mL, and 45%, for serum ferritin and transferrin saturation, respectively. The reference ranges for males are 300 ng/mL, and 50%, for serum ferritin and transferrin saturation, respectively. As the serum ferritin concentration range was very wide, serum ferritin concentrations above 1650 ng/mL are not shown. Abbreviations: + / + : individuals with no C282Y or H63D mutation in *HFE* gene; C282Y/ + : *HFE* C282Y heterozygosity; C282Y/C282Y: *HFE* C282Y homozygosity; H63D/ + : *HFE* H63D heterozygosity; H63D/H63D: *HFE* H63D homozygosity; C282Y/H63D: *HFE* compound heterozygosity. Number of individuals present in each category: female + / + (n = 128); male + / + (n = 119); female C282Y/ + (n = 180); male C282Y/ + (n = 146); female C282Y/C282Y (n = 141); male C282Y/C282Y (n = 75); female H63D/ + (n = 43); male H63D/ + (n = 36); female H63D/H63D (n = 9); male H63D/H63D (n = 8); female C282Y/H63D (n = 41); male C282Y/H63D (n = 27). The data plotted in these figures correspond to the raw data, and was not imputed. Thus, these figures represent the data of 953 individuals for which a genotype was available.

Serum ferritin levels (Fig. 2a) were increased in males, especially for both types of *HFE* homozygosity (C282Y and H63D), with the median value being dramatically above the reference range. More than 75% of females from each genotype had serum ferritin levels below the female reference range, with the exception of female C282Y homozygotes.

Similarly, transferrin saturation levels (Fig. 2b) were also increased in males. Both male *HFE* homozygotes for C282Y and H63D, as well as male compound heterozygotes showed median values above the reference range. In contrast, only female C282Y homozygotes had median values above the reference range.

In contrast to the previous results, uibc levels (Fig. 2c) were decreased in males. Additionally, female and male *HFE* C282Y homozygotes had lower uibc levels when compared to the other genotypes.

**Figure 3:**
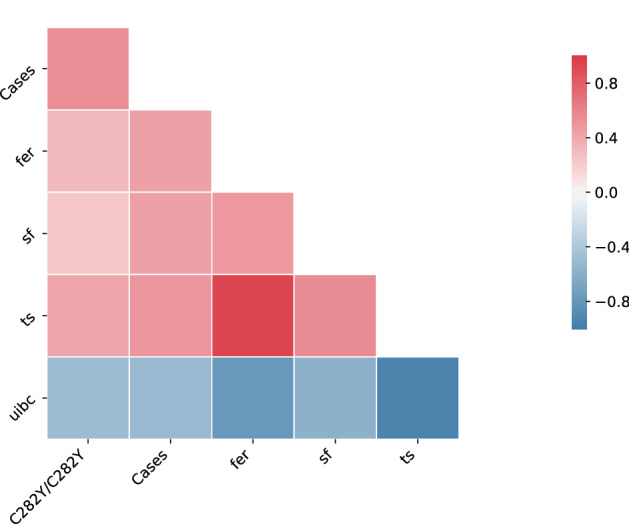
Spearman correlation plot of HH associated variables from the family dataset. Variables with more than 10% of missing values were removed. Only risk factors which fulfil the following 2 criteria are shown: (1) absolute correlation with the target variable (Cases) equal or larger than 0.4 and (2) Bonferroni corrected p-value ≤ 0.05. Abbreviations: C282Y/C282Y: *HFE* C282Y homozygosity; fer: serum iron concentration; sf: serum ferritin concentration; ts: transferrin saturation; uibc: unsaturated iron binding capacity.

In the next step, Spearman correlation was calculated in order to identify which variables were correlated (Supplementary Table 1). A threshold of 0.4 was used to extract those variables, which exhibited an absolute correlation larger or equal to this value with the variable ‘Cases’. Only five variables (*HFE* C282Y homozygosity, serum iron concentration, serum ferritin concentration, transferrin saturation, and uibc) showed an absolute correlation equal or larger than 0.4 with the target variable (Fig. 3). Among those, only three (*HFE* C282Y homozygosity, transferrin saturation, and uibc) had an absolute correlation equal or larger than 0.5 with the target variable. As shown in Fig. 3, uibc was negatively correlated with all the other variables, with the largest negative correlation being observed with transferrin saturation (r = − 0.936). On the other hand, transferrin saturation and serum iron concentration showed the largest positive correlation (r = 0.938).

### Identification of the most relevant risk factors for HH

Six different methods were employed to determine the relevance of variables in the discrimination between HH cases and healthy individuals, and the results are shown in Table 2. Each tested method produced different results, i.e., the rate of overlapping variables present in all the six lists was less than 25%. Only six risk factors out of 28 (age, serum iron concentration, serum ferritin concentration, transferrin saturation, uibc, and *HFE* C282Y homozygosity) were present in all the six lists. Other risk factors, such as alanine aminotransferase serum activity or mean corpuscular volume, were retrieved in five out of the six lists.

When considering only the top five risk factors, we observed that serum ferritin concentration, transferrin saturation, uibc, and *HFE* C282Y homozygosity were ranked as the most important variables among all the methods. Serum iron concentration, and gender were ranked four, and two times out of six feature ranking methods, respectively, as top five risk factors.

Family health history of haemochromatosis (number of relatives affected by haemochromatosis or a positive family health history of haemochromatosis) was ranked as a significant risk factor in two out of the six ranking methods. Additionally, family health history was not ranked in the top 5 nor in the top 10 risk factors, suggesting that in this dataset this variable might not be so relevant or the data were not sufficiently collected.

A literature review of the variables in Table 2 was performed to validate the results, and most of these risk factors have been previously associated with HH/iron overload. However, new statistical significant associations not previously reported have been identified (i.e., red blood cell distribution width, haemoglobin, haematocrit and gamma glutamyl transferase serum activity).

Thus, in order to comprehensively select the most significant and consistent risk factors, different sets of risk factors were selected, as described in the Feature selection subsection. This experiment was designed to evaluate which one would provide the best performance in the risk prediction of HH.

1. Set A includes all the risk factors obtained by method A.
2. Set B includes all the risk factors obtained by method B (see Supplementary Fig. 1).
3. Set C includes all the risk factors obtained by method C.
4. Set A&B includes all the common risk factors obtained by methods A and B: age, serum iron concentration, serum ferritin concentration, transferrin saturation, uibc, *HFE* C282Y homozygosity, and mean corpuscular volume.
5. Set A&C includes all the common risk factors obtained by methods A and C: age, serum iron concentration, serum ferritin concentration, transferrin saturation, uibc, *HFE* C282Y homozygosity, and total iron binding capacity.
6. Set B&C includes all the common risk factors obtained by methods B and C: age, serum iron concentration, serum ferritin concentration, transferrin saturation, uibc, *HFE* C282Y homozygosity, and alanine aminotransferase serum activity.
7. Set A&B&C includes all the common risk factors between the methods A, B and C: age, serum iron concentration, serum ferritin concentration, transferrin saturation, uibc, and *HFE* C282Y homozygosity.
8. Set ALL: no feature selection was performed and all features (n = 122) available in the processed dataset were used to train a ML classifier.

### Evaluation of the ML models

After selecting eight different sets of risk factors for HH (as described in the Feature selection subsection), we tested seven popular machine learning algorithms to determine which combination of risk factors and algorithm provided the best performance in the diagnosis of HH. The results of this evaluation are shown in Table 3. Of all tested algorithms, XGB combined with set B (n = 13 risk factors: age, gender, serum iron concentration, serum ferritin concentration, transferrin saturation, uibc, *HFE* C282Y homozygosity, mean corpuscular volume, Asian ethnicity, number of relatives affected by haemochromatosis, red blood cell count, alanine aminotransferase serum activity, and aspartate aminotransferase serum activity) provided the best performance (F1 score = 0.8095 ± 0.069), and was the best algorithm for most all the risk factors sets evaluated. Interestingly, the different sets and combinations of risk factors provided similar performance values, except set A and set ALL, whose performance slightly decreased. Finally, the standard deviation of the different performance metrics was wide on the test set, but very narrow on the validation set (Supplementary Table 2).

**Table 3:** Hereditary haemochromatosis risk score model’s performance on the test set.

Set	Number of features	Model	Accuracy ± sd	F1 Score ± sd	Sensitivity ± sd	Specificity ± sd
A	15	XGB	0.8797 ± 0.0383	0.7772 ± 0.0654	0.784 ± 0.0815	0.9145 ± 0.0496
		RF	0.8692 ± 0.0405	0.7489 ± 0.0697	0.7285 ± 0.0741	0.9202 ± 0.0465
		LR	0.8534 ± 0.0548	0.7465 ± 0.0749	0.7912 ± 0.0644	0.8759 ± 0.0764
B	13	XGB	0.8995 ± 0.0376	0.8095 ± 0.0691	0.8032 ± 0.0985	0.9344 ± 0.0467
		MLP	0.8817 ± 0.0387	0.7766 ± 0.0645	0.7638 ± 0.0661	0.9244 ± 0.0512
		RF	0.8838 ± 0.042	0.7762 ± 0.0861	0.7638 ± 0.1133	0.9273 ± 0.0453
C	9	XGB	0.8974 ± 0.0404	0.8092 ± 0.0756	0.8234 ± 0.1136	0.9244 ± 0.0456
		RF	0.889 ± 0.0495	0.7863 ± 0.0923	0.7683 ± 0.1089	0.9329 ± 0.0457
		KNN	0.8796 ± 0.0342	0.7742 ± 0.0645	0.7825 ± 0.1088	0.9145 ± 0.046
A&B	7	XGB	0.8943 ± 0.0389	0.8041 ± 0.0704	0.8154 ± 0.0989	0.923 ± 0.0456
		MLP	0.8764 ± 0.0557	0.7776 ± 0.0936	0.8031 ± 0.0973	0.903 ± 0.0577
		RF	0.8786 ± 0.0426	0.7746 ± 0.0788	0.7838 ± 0.0944	0.913 ± 0.0443
A&C	7	RF	0.8901 ± 0.0283	0.798 ± 0.0429	0.8111 ± 0.068	0.9187 ± 0.0466
		XGB	0.8849 ± 0.0462	0.786 ± 0.0841	0.7954 ± 0.1071	0.9173 ± 0.0493
		MLP	0.8754 ± 0.0442	0.7769 ± 0.0771	0.8112 ± 0.094	0.8988 ± 0.0499
B&C	7	XGB	0.8974 ± 0.0373	0.8067 ± 0.0675	0.804 ± 0.1021	0.9316 ± 0.0511
		KNN	0.8838 ± 0.039	0.7921 ± 0.0593	0.8185 ± 0.0598	0.9073 ± 0.057
		MLP	0.8796 ± 0.0491	0.7803 ± 0.0858	0.7992 ± 0.1039	0.9087 ± 0.0575
A&B&C	6	XGB	0.8891 ± 0.0397	0.7939 ± 0.0738	0.8034 ± 0.0937	0.9202 ± 0.0426
		RF	0.8807 ± 0.0417	0.7793 ± 0.0734	0.7877 ± 0.0871	0.9145 ± 0.0507
		MLP	0.8764 ± 0.0493	0.778 ± 0.0862	0.8112 ± 0.1131	0.9002 ± 0.0586
ALL	122	XGB	0.8848 ± 0.0431	0.7818 ± 0.0809	0.7798 ± 0.1157	0.923 ± 0.0522
		RF	0.8649 ± 0.0486	0.7265 ± 0.0954	0.6729 ± 0.0941	0.9344 ± 0.0475
		LR	0.8408 ± 0.0557	0.7211 ± 0.0711	0.76 ± 0.0807	0.8701 ± 0.0784

Only the top three algorithms are shown for each feature set.

*sd* standard deviation.

**Figure 4:**
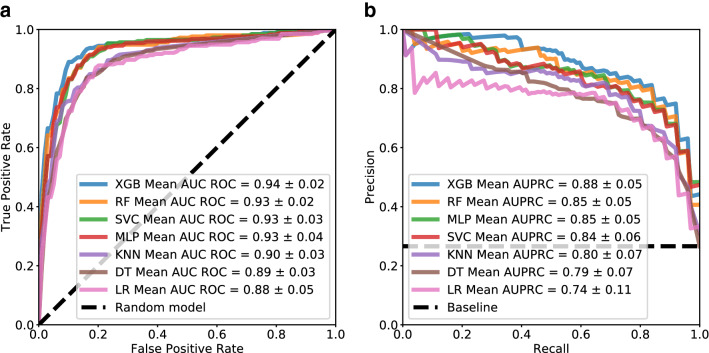
Performance curves for the best set of risk factors. (**a**) Receiver Operating Characteristic (ROC) curve. The ROC curve was interpolated with the function interp from the *scipy* package using the true and the false positive rate values obtained in each CV run. (**b**) Precision-Recall curve. The PR curve was interpolated with the function interp from the *scipy* package using the recall and precision values obtained in each CV run.

The Receiver Operating Characteristic (ROC) and the Precision-Recall (PR) curves for the best set of risk factors (set B) are depicted in Fig. 4. The area of the ROC and PR curves allow further assessment of the performance of the models. While AUCROC is useful in assessing performance of balanced data, AUPRC is best suited to tackle and evaluate the performance of imbalanced data. The best classifier obtained 0.94 ± 0.02 AUCROC and 0.88 ± 0.05 AUPRC for tenfold stratified CV. The obtained results demonstrated that our model performed well with imbalanced data.

After identifying the best HH risk score model, we optimised the hyperparameters of XGB algorithm (*xgboost* package) and employed a tenfold CV GridSearch approach on the whole dataset, resulting on the following optimized hyperparameters: max_depth = 3, min_child_weight = 4, gamma = 0, colsample_bytree = 0.8, subsample = 0.75, reg_alpha = 1e−05, and n_estimators = 787.

### Model benchmarking

After evaluating which combination of risk factors and algorithm provided the best performance, we concluded that the usage of thirteen risk factors (age, gender, serum iron concentration, serum ferritin concentration, transferrin saturation, uibc, *HFE* C282Y homozygosity, mean corpuscular volume, Asian ethnicity, number of relatives affected by haemochromatosis, red blood cell count, alanine aminotransferase serum activity, and aspartate aminotransferase serum activity) as an input dataset for an XGB classifier provided the most accurate results in the risk prediction of HH.

To date, only one readily available tool, the IRON score, was proposed to help in the screening of iron overload. We evaluated how the new proposed model compared to the IRON score. The new disease risk model was benchmarked to the IRON score using two different approaches, per the below:

1. The first one was to apply the IRON score to the family study dataset. As no threshold was provided in the IRON score, five different ones were tested, similarly to Mainous et al*.*
2. As a second approach, we used the IRON score risk factors (age, gender, medical history of liver condition, osteoporosis and thyroid disease) as input parameters to train different machine learning algorithms, see Table 4.

**Table 4:** Performance of the IRON score and risk factors (age, gender, medical history of liver condition, osteoporosis and thyroid disease) on the family study from the HEIRS cohort.

	Number of features	Model/criterion	F1 Score ± sd	AUCROC ± sd	AUPRC ± sd
IRON score risk factors	5	RF	0.2016 ± 0.0606	0.6041 ± 0.0588	0.356 ± 0.0576
		KNN	0.1937 ± 0.0701	0.5637 ± 0.0565	0.313 ± 0.0512
		SVC	0.1931 ± 0.0935	0.4903 ± 0.0901	0.2912 ± 0.0544
IRON Score	5	> 0	0.4540	0.5744	0.2990
		> 2	0.4257	0.5594	0.2924
		> 3	0.4031	0.5475	0.2869
		> 5	0.3767	0.5309	0.2792
		> 6	0.3202	0.5375	0.2837

Only the top three algorithms are shown for the IRON score feature set.

*AUCROC* area under the ROC curve, *AUPRC* area under the precision-recall curve, *sd* standard deviation.

When comparing the performance (i.e., F1 score) of the new proposed model with the IRON score, our model (F1 score = 0.8095 ± 0.0691) outperformed the latter (F1 score between 0.3202 and 0.4540) by at least 35%, depending on the threshold selected. Furthermore, the XGB classifier trained with the risk factors selected by our approach (F1 score = 0.8095 ± 0.0691) outperformed all the classifiers trained with the IRON score risk factors (F1 score = 0.2016 ± 0.0606).

We further compared the AUCROC of the different algorithms trained using our pre-selected risk factors or the risk factors from the IRON score. We confirmed that the AUCROC of the new HH risk model (AUCROC = 0.94 ± 0.02) outperformed the best model obtained on the risk factors from the IRON score (AUCROC = 0.6041 ± 0.0588).

Finally, we compared the AUCROC values of the best HH screening model (XGB and risk factors set B) with the other ones (including the IRON score feature set) in order to evaluate whether its performance was significantly different. We could confirm that all the models trained on the IRON score feature set were statistically different from the best HH screening model, as well as three models trained on the full set of features (set ALL) (Table 5).

**Table 5:** Statistical comparison of the AUCROC values between the best HH risk score model (XGB and feature set B) and all the other tested models.

Model	Feature set	Adjusted p-value
DT	IRON score	0.0099
KNN	IRON score	0.0099
LR	IRON score	0.0099
MLP	IRON score	0.0099
RF	IRON score	0.0099
SVC	IRON score	0.0099
XGB	IRON score	0.0099
DT	ALL	0.0099
KNN	ALL	0.0240
MLP	ALL	0.0240

In this table are listed tested models which yielded statistically significant lower AUCROC values when compared to the best HH risk score model. The models performance were compared using 2-tail Wilcoxon signed-rank test followed by Bonferroni correction. Only models with an adjusted p-value ≤ 0.05 are shown.

To conclude, the HH screening model proposed in this work has a higher performance when compared to the IRON score.

## Discussion

Improved disease risk stratification models promise to aid in the diagnosis and prevention of diseases, such as diabetes, HH or cardiovascular disease. In this study, we have identified the most relevant risk factors for HH in the family study of the HEIRS cohort and proposed a new disease risk screening model for this disease.

It is important to note that even though we used the family study data from the HEIRS cohort, our definition of “cases” differs from the one captured within the HEIRS study. In our definition, *HFE* C282Y homozygous were not considered true cases, as not all *HFE* C282Y homozygous will develop HH due to the low penetrance of this genotype^1,31^. As described in the Data preprocessing subsection, “cases” were defined as individuals having a provisional classification or a positive personal history of haemochromatosis.

We investigated the characteristics of the family study cohort, and as expected, HH cases had median serum ferritin and transferrin saturation levels above the reference levels used for HH diagnosis^23,24^. Commonly accepted reference ranges for females are 200 ng/mL, and 45%, for serum ferritin and transferrin saturation, respectively, and for males are 300 ng/mL, and 50%, for serum ferritin and transferrin saturation, respectively. Additionally when considering the individual's genotype, *HFE* C282Y homozygous had a higher penetrance of iron overload. This was further validated by the observed positive correlation between these variables and being an HH case. Furthermore, we observed that there were almost as many cases as healthy individuals of Asian/Pacific Islander descent. Interestingly, one of the feature ranking methods (XGB with RFE stratified tenfold CV) identified Asian/Pacific Islander background as one of the top risk factors for HH. Previous studies on the HEIRS cohort have shown that Asian/Pacific Islander individuals had a high prevalence of elevated serum ferritin and transferrin saturation levels^21^.

Several feature ranking methods were tested and the results showed that the list of the most important risk factors varied considerably among the methods. In total, only six risk factors out of 28 unique ones were retrieved by all tested feature ranking methods, representing a total of 21.4%. This low percentage of overlap demonstrates the lack of consistency between the methods. Statistical methods, and RFE using CV provided a consistent set of risk factors at each run without the need to impose a threshold. However, the other tested methods (mutual information or RFE without CV) showed inconsistent results after every run. In order to avoid this, a random seed was selected. Another drawback is the need to select a threshold in order to extract the most relevant variables, i.e., the top 10 or top 15 risk factors. In this study, we considered the top 15 risk factors as the most significant, to be consistent with the number of risk factors returned by the statistical method. Interestingly, if we had considered only the top 5 risk factors, we observed that these variables were quite consistent among the methods. These results demonstrate that feature selection needs to be performed carefully, depending on the method selected and on the threshold used, the final list of the most significant risk factors will vary. Based on these results, we suggest that feature ranking methods based on mutual information or RFE without CV should be applied with caution. Additionally, these results demonstrate that the most significant and robust risk factors are ranked higher on the list for all feature ranking methods.

The onset of HH clinical symptoms have been reported to be age and gender dependent. While men remain asymptomatic until the age of 30–40, women start having symptoms after the age of 40–50^30^, once they enter menopause. Interestingly, age was identified by all feature ranking methods and appeared to be a very consistent risk factor. Accordingly, the results demonstrated that HH cases were mostly older than 40 years old, with the highest HH prevalence observed in individuals from 60 to 69 years old. On the other hand, gender was only found by two out of the six methods, suggesting that for this dataset gender might not be such a determinant factor.

Serum iron and ferritin concentration, transferrin saturation, and *HFE* C282Y homozygosity were found by all feature ranking methods, and also showed a high positive correlation with HH cases. It has been reported that 80 to 85% of HH cases are *HFE* C282Y homozygous^1^. Furthermore, serum ferritin concentration and transferrin saturation are common first line diagnostic markers for HH^23,24^. These results demonstrate the validity of the feature ranking approach.

Two other risk factors (uibc and mean corpuscular volume) were also identified by feature ranking. uibc has been reported to be decreased in individuals with iron overload^32^ and is equally good as transferrin saturation in the identification of *HFE* haemochromatosis^21,22^. On the other hand, mean corpuscular volume has been reported to be increased in HH individuals, and could be also used to screen HH individuals^29^.

Two additional risk factors alt and ast have been identified by the different feature ranking methods, and have been previously reported to be mildly abnormal in a sample of 100 HH individuals^27^. Additionally, in the *HFE* C282Y homozygotes subpopulation from the HEIRS cohort, alt has been shown to be higher when comparing previously diagnosed and newly diagnosed homozygotes with elevated SF to newly diagnosed homozygotes with normal SF. Even though alt and ast were not statistically significant between cases and controls in the HEIRS family study, they were ranked in 5, and 3 out of the 6 tested feature ranking methods, respectively. These results suggest that alone, their prediction power is very low. However, when in combination with other risk factors, they might become more relevant.

This approach could also identify statistically significant risk factors such as red blood cell distribution width, haemoglobin, haematocrit and gamma glutamyl transferase serum activity which have not been previously reported in the literature. Red blood cell distribution width, haemoglobin, and haematocrit are iron related markers and could be further investigated in the future. A previous study on the African American population of the HEIRS cohort has shown that gamma glutamyl transferase serum activity is positively correlated with serum ferritin and transferrin saturation levels^9^. In our analysis, we included all ethnic backgrounds, and around 76% of the individuals were Caucasian. These results suggest that similar associations occur in the HEIRS family study.

One of our hypotheses was that the integration of different sources of data, i.e., family health history of HH or clinical data, would increase the performance of HH risk screening models. However, in the family study of the HEIRS cohort, family health history was ranked very poorly, and retrieved in only two out of the six tested methods. This could be explained by the fact that this dataset might be biased. In the future, it would be of value to test if our hypothesis holds true in a general population cohort. Regardless, we could demonstrate that the integration of demographic information (i.e., age), laboratory blood values (i.e., uibc, mean corpuscular volume and other biomarkers), the number of relatives with a HH medical history, and the individual's genotype led to an improvement in the performance of the proposed HH screening model.

Finally, the best found model was benchmarked against the IRON score using two approaches and we demonstrated that our risk score model (AUCROC = 0.94 ± 0.02) outperforms it. The two benchmarking approaches included (1) directly applying the IRON score to the HEIRS family cohort, and (2) extracting the IRON score risk factors to train a machine learning-based model. Both approaches yielded very low performance results, when compared to the other risk stratification models tested in our pipeline. Previous results have shown that the IRON score obtained 0.72, and 0.685 AUCROC for the training, and test sets, respectively. When applied to the HEIRS family cohort, the obtained AUCROC values were lower. This could be explained by the fact that the populations used for the training of the IRON score are very different from the HEIRS family study. On the one hand, the IRON score^13^ was trained and validated on a representative sample of the US population (NHANES) and was developed for general primary care screening. On the other hand, we trained all the models on a US subpopulation (composed of cases and their family members) known to be at high risk of haemochromatosis.

Other risk score models based only on clinical symptoms, or integrating these with laboratory data have recently been proposed^14,15^. As we did not have access to these models, including the script, model parameters, HPO terms, laboratory variables and ICD-codes, we were unable to replicate and to test them on the family study of the HEIRS cohort. The PheRS was trained on the Vanderbilt University Medical Center Synthetic Derivative dataset which is a de-identified electronic health database of about 2.5 M individuals. The results available on the original study have demonstrated that the PheRS achieved a maximum 0.85 AUCROC, suggesting that our proposed HH risk score model yields similar performance results.

When comparing the number of cases versus controls among the different datasets, we observed that our model, the IRON score, and the PheRS, were class imbalanced in a ratio of ~ 1:3 (cases:controls), ~ 1:87 (cases:controls), and ~ 1/6234 (cases:controls), respectively. In our model, we tried to address the problem of imbalanced data by using stratified cross-validation in order to ensure that there would be a similar number of cases on each fold. In addition, we also calculated the AUPRC performance metric which is more reliable than the AUCROC it-self when in presence of imbalanced data.

Both the IRON score and the PheRS were tested on an external validation set, which is one of the main limitations of this work. To our knowledge, only the HEIRS cohort has explored and captured the family dimension. Thus, a similar HH family study on another cohort is not currently available, which brings difficulties in the external model validation. A second limitation is caused by the problem of class imbalance. We observed that specificity and sensitivity values were not balanced. Additionally some models yielded a large standard deviation. This could be explained by the class imbalance (1:3) and also by the way how cases were defined. Cases were defined as in the original HEIRS cohort and as individuals with a HH personal medical history. Even though the latter are known to suffer from HH, some of them might have already started HH treatment and their blood values might be already in a healthy range. Thus, these cases will be extremely difficult to predict and lead to large deviations of the model’s performance. In the future, the problem of class imbalance could be tackled by employing SMOTE or sampling methods.

Here, we propose a new risk stratification model for HH based on 13 easily obtainable predictive markers. However, we observed that employing a subset of these risk factors (i.e. Sets A&B or B&C) or even Set C led to similar performance results. As these sets of risk factors were not validated by a clinical expert, here, we reported the best set found by the machine learning algorithms. In the future, these sets should be clinically validated and the risk stratification model could be translated to a user-friendly application. This tool would provide an easy method for medical doctors to screen their patients in the clinical practice and prevent the onset of the symptoms caused by chronic iron overload. Furthermore, the ML pipeline developed in this study accepts a flexible number of input risk factors and will provide an easy framework for the scientific community to develop risk stratification models for any disease of interest, such as diabetes, hypercholesterolemia or any type of cancer.

## Supplementary information

Supplementary Information

## Data Availability

The HEIRS cohort is available upon application at NHLBI. All the code will be available upon request.
