# Supplementary material for: An efficient machine learning-based approach for screening individuals at risk of hereditary haemochromatosis
Source: Sci Rep. 2020 Nov 26;10:20613. doi: 10.1038/s41598-020-77367-6 (PMC7691515; doi:10.1038/s41598-020-77367-6)
Supplement: Supplementary file 1 — Supplementary Information [file 41598_2020_77367_MOESM1_ESM.pdf]

# Supplementary information

## An efficient machine learning-based approach for screening individuals at risk of hereditary haemochromatosis

Patricia Martins Conde[1,2], Thomas Sauter[2], Thanh Phuong Nguyen[1,2,*]

[1] Megeno S.A, Esch-sur-Alzette, Luxembourg

[2] University of Luxembourg, Esch-sur-Alzette, Luxembourg

[*] Corresponding author: phuong.nguyen@megeno.com

**Supplementary table 1.** Spearman correlation of Hereditary Haemochromatosis (HH) associated variables from the family dataset of the HEIRS cohort. Only variables, which fulfil the following 2 criteria are shown: 1) absolute correlation equal or larger than 0.3 and 2) Bonferroni corrected p-value equal or smaller than 0.05. The encoding of the categorical variables was performed as follows. Gender: females were encoded as 1 and males as 0. Cases: HH cases were encoded as 1, and healthy individuals as 0. Individuals at menopause were encoded as 1, and the ones not yet at menopause were encoded as 0. Males were considered to be at menopause.

| | Cases | | Serum ferritin | | Transferrin saturation | |
|---|---|---|---|---|---|---|
| | r | Adj. p-value | r | Adj. p-value | r | Adj. p-value |
| alt | 0.0413 | 0.3906 | 0.3974 | <0.0001 | 0.1324 | 0.0002 |
| ast | 0.0454 | 0.3378 | 0.3168 | <0.0001 | 0.1539 | <0.0001 |
| C282Y/+ | -0.3234 | <0.0001 | -0.2350 | <0.0001 | -0.2346 | <0.0001 |
| C282Y/C282Y | 0.5356 | <0.0001 | 0.2405 | <0.0001 | 0.4097 | <0.0001 |
| Cases | - | - | 0.4379 | <0.0001 | 0.5041 | <0.0001 |
| Gender | -0.0499 | 0.2787 | -0.4085 | <0.0001 | -0.1582 | <0.0001 |
| fer | 0.4406 | <0.0001 | 0.4847 | <0.0001 | 0.9381 | <0.0001 |
| ggt | 0.1166 | 0.0019 | 0.3783 | <0.0001 | 0.1161 | 0.0013 |
| hct | 0.1342 | 0.0002 | 0.4086 | <0.0001 | 0.2951 | <0.0001 |
| hgb | 0.1486 | <0.0001 | 0.4382 | <0.0001 | 0.3372 | <0.0001 |
| mch | 0.2950 | <0.0001 | 0.3402 | <0.0001 | 0.4677 | <0.0001 |
| mcv | 0.2921 | <0.0001 | 0.3010 | <0.0001 | 0.4333 | <0.0001 |
| rdw | -0.1967 | <0.0001 | -0.2855 | <0.0001 | -0.3921 | <0.0001 |
| rhMen | 0.1715 | <0.0001 | 0.4314 | <0.0001 | 0.1566 | <0.0001 |
| sf | 0.4379 | <0.0001 | - | - | 0.5591 | <0.0001 |
| tibc | -0.3556 | <0.0001 | -0.4284 | <0.0001 | -0.5479 | <0.0001 |
| ts | 0.5041 | <0.0001 | 0.5591 | <0.0001 | - | - |
| uibc | -0.5117 | <0.0001 | -0.5744 | <0.0001 | -0.9364 | <0.0001 |

Abbreviations: alt: alanine aminotransferase serum activity; ast: aspartate aminotransferase serum activity; C282Y/+: *HFE* C282Y heterozygosity; C282Y/C282Y: *HFE* C282Y homozygosity; fer: serum iron concentration; ggt: gamma glutamyl transferase serum activity; hct: haematocrit; hgb: haemoglobin concentration; mch: mean corpuscular haemoglobin/RBC; mcv: mean corpuscular volume; rdw: red blood cell distribution width; rhMen: at menopause; sf: serum ferritin concentration; tibc: total iron binding capacity; ts: transferrin saturation; uibc: unsaturated iron binding capacity.

**Supplementary table 2.** HH risk score model's performance. In grey is highlighted the best risk score model for HH. Abbreviation: sd=standard deviation.

| Set | Number of features | Model | Accuracy ± sd | | F1 Score ± sd | | Sensitivity ± sd | | Specificity ± sd | |
|---|---|---|---|---|---|---|---|---|---|---|
| | | | Validation set | Test set | Validation set | Test set | Validation set | Test set | Validation set | Test set |
| A | 15 | XGB | 0.9417 ± 0.0065 | 0.8797 ± 0.0383 | 0.8928 ± 0.0111 | 0.7772 ± 0.0654 | 0.9116 ± 0.0079 | 0.784 ± 0.0815 | 0.9526 ± 0.0071 | 0.9145 ± 0.0496 |
| | | RF | 0.9565 ± 0.0183 | 0.8692 ± 0.0405 | 0.9185 ± 0.0338 | 0.7489 ± 0.0697 | 0.9182 ± 0.0264 | 0.7285 ± 0.0741 | 0.9704 ± 0.0159 | 0.9202 ± 0.0465 |
| | | LR | 0.8593 ± 0.0075 | 0.8534 ± 0.0548 | 0.7537 ± 0.0118 | 0.7465 ± 0.0749 | 0.8092 ± 0.024 | 0.7912 ± 0.0644 | 0.8775 ± 0.013 | 0.8759 ± 0.0764 |
| | | MLP | 0.9254 ± 0.0158 | 0.8597 ± 0.0576 | 0.866 ± 0.0291 | 0.7462 ± 0.0938 | 0.9073 ± 0.0406 | 0.7632 ± 0.0822 | 0.932 ± 0.0119 | 0.8945 ± 0.0614 |
| | | SVC | 0.9028 ± 0.0115 | 0.8566 ± 0.0526 | 0.8169 ± 0.0247 | 0.72 ± 0.1134 | 0.8167 ± 0.0356 | 0.7045 ± 0.1415 | 0.9341 ± 0.0045 | 0.9116 ± 0.0545 |
| | | DT | 0.8985 ± 0.0221 | 0.8493 ± 0.0533 | 0.7999 ± 0.0446 | 0.7025 ± 0.0983 | 0.7637 ± 0.0502 | 0.666 ± 0.1009 | 0.9474 ± 0.0165 | 0.9159 ± 0.0522 |
| | | KNN | 0.9873 ± 0.0401 | 0.8471 ± 0.0535 | 0.9748 ± 0.0796 | 0.7006 ± 0.0979 | 0.9711 ± 0.0915 | 0.6726 ± 0.1167 | 0.9932 ± 0.0215 | 0.9102 ± 0.0645 |
| B | 13 | XGB | 0.9621 ± 0.007 | 0.8995 ± 0.0376 | 0.928 ± 0.0132 | 0.8095 ± 0.0691 | 0.9191 ± 0.0145 | 0.8032 ± 0.0985 | 0.9776 ± 0.0056 | 0.9344 ± 0.0467 |
| | | MLP | 0.9283 ± 0.0202 | 0.8817 ± 0.0387 | 0.865 ± 0.0382 | 0.7766 ± 0.0645 | 0.8626 ± 0.0384 | 0.7638 ± 0.0661 | 0.9521 ± 0.0153 | 0.9244 ± 0.0512 |
| | | RF | 0.9647 ± 0.0175 | 0.8838 ± 0.042 | 0.9328 ± 0.0331 | 0.7762 ± 0.0861 | 0.9195 ± 0.0312 | 0.7638 ± 0.1133 | 0.9811 ± 0.0132 | 0.9273 ± 0.0453 |
| | | SVC | 0.9257 ± 0.0088 | 0.8786 ± 0.0412 | 0.8594 ± 0.0165 | 0.7689 ± 0.0802 | 0.8543 ± 0.0199 | 0.7642 ± 0.1125 | 0.9515 ± 0.0082 | 0.9201 ± 0.0487 |
| | | KNN | 0.9789 ± 0.0444 | 0.8681 ± 0.0376 | 0.9584 ± 0.0877 | 0.7411 ± 0.0601 | 0.951 ± 0.1034 | 0.7009 ± 0.0525 | 0.9891 ± 0.0231 | 0.9287 ± 0.0504 |
| | | LR | 0.85 ± 0.0077 | 0.8451 ± 0.0544 | 0.7397 ± 0.0122 | 0.7321 ± 0.0833 | 0.8014 ± 0.0282 | 0.7914 ± 0.1147 | 0.8677 ± 0.0141 | 0.8644 ± 0.0692 |
| | | DT | 0.9201 ± 0.018 | 0.8514 ± 0.0338 | 0.8419 ± 0.037 | 0.7027 ± 0.0708 | 0.8019 ± 0.0446 | 0.6655 ± 0.0946 | 0.9629 ± 0.0107 | 0.9187 ± 0.0342 |
| C | 9 | XGB | 0.9538 ± 0.0131 | 0.8974 ± 0.0404 | 0.9128 ± 0.0248 | 0.8092 ± 0.0756 | 0.9081 ± 0.0237 | 0.8234 ± 0.1136 | 0.9704 ± 0.01 | 0.9244 ± 0.0456 |
| | | RF | 0.9636 ± 0.0131 | 0.889 ± 0.0495 | 0.9313 ± 0.0244 | 0.7863 ± 0.0923 | 0.9265 ± 0.0215 | 0.7683 ± 0.1089 | 0.977 ± 0.0104 | 0.9329 ± 0.0457 |
| | | KNN | 0.9901 ± 0.0313 | 0.8796 ± 0.0342 | 0.9816 ± 0.0581 | 0.7742 ± 0.0645 | 0.9829 ± 0.0541 | 0.7825 ± 0.1088 | 0.9927 ± 0.0231 | 0.9145 ± 0.046 |
| | | SVC | 0.9047 ± 0.0036 | 0.8774 ± 0.0468 | 0.8217 ± 0.008 | 0.7696 ± 0.0918 | 0.8259 ± 0.0151 | 0.7752 ± 0.1255 | 0.9333 ± 0.0033 | 0.9144 ± 0.0543 |
| | | MLP | 0.9173 ± 0.0122 | 0.8712 ± 0.0431 | 0.8495 ± 0.0244 | 0.7652 ± 0.0697 | 0.8797 ± 0.0381 | 0.7797 ± 0.0845 | 0.9309 ± 0.0046 | 0.9045 ± 0.0616 |
| | | LR | 0.8558 ± 0.0067 | 0.8534 ± 0.0547 | 0.7524 ± 0.0097 | 0.7521 ± 0.0813 | 0.8233 ± 0.0114 | 0.8228 ± 0.0894 | 0.8676 ± 0.0087 | 0.8645 ± 0.07 |
| | | DT | 0.9129 ± 0.014 | 0.8587 ± 0.0388 | 0.8341 ± 0.0258 | 0.7302 ± 0.0809 | 0.8228 ± 0.0277 | 0.7289 ± 0.1277 | 0.9455 ± 0.0148 | 0.906 ± 0.051 |

**Continue on next page**

| Set | Number of features | Model | Accuracy ± sd | | F1 Score ± sd | | Sensitivity ± sd | | Specificity ± sd | |
|---|---|---|---|---|---|---|---|---|---|---|
| | | | Validation set | Test set | Validation set | Test set | Validation set | Test set | Validation set | Test set |
| A&B | 7 | **XGB** | 0.9284 ± 0.009 | 0.8943 ± 0.0389 | 0.8689 ± 0.0163 | 0.8041 ± 0.0704 | 0.8915 ± 0.0155 | 0.8154 ± 0.0989 | 0.9418 ± 0.007 | 0.923 ± 0.0456 |
| | | **MLP** | 0.8981 ± 0.006 | 0.8764 ± 0.0557 | 0.8149 ± 0.0102 | 0.7776 ± 0.0936 | 0.8434 ± 0.0122 | 0.8031 ± 0.0973 | 0.9179 ± 0.0072 | 0.903 ± 0.0577 |
| | | **RF** | 0.9454 ± 0.0177 | 0.8786 ± 0.0426 | 0.8986 ± 0.0328 | 0.7746 ± 0.0788 | 0.9077 ± 0.0319 | 0.7838 ± 0.0944 | 0.9591 ± 0.0149 | 0.913 ± 0.0443 |
| | | **SVC** | 0.8873 ± 0.0053 | 0.8691 ± 0.0497 | 0.79 ± 0.0092 | 0.7583 ± 0.0893 | 0.7975 ± 0.0149 | 0.7675 ± 0.098 | 0.9198 ± 0.0074 | 0.9059 ± 0.0551 |
| | | **DT** | 0.891 ± 0.0068 | 0.8691 ± 0.0352 | 0.7905 ± 0.0197 | 0.7441 ± 0.0723 | 0.7761 ± 0.0492 | 0.7172 ± 0.0923 | 0.9326 ± 0.0131 | 0.9244 ± 0.0426 |
| | | **KNN** | 0.9389 ± 0.0531 | 0.8618 ± 0.059 | 0.8857 ± 0.0993 | 0.7416 ± 0.1044 | 0.8884 ± 0.0976 | 0.7435 ± 0.1208 | 0.9572 ± 0.0374 | 0.9045 ± 0.064 |
| | | **LR** | 0.8483 ± 0.0066 | 0.8471 ± 0.0565 | 0.7398 ± 0.0108 | 0.7411 ± 0.0833 | 0.811 ± 0.0123 | 0.8111 ± 0.0903 | 0.8618 ± 0.0062 | 0.8602 ± 0.0673 |
| A&C | 7 | **RF** | 0.9344 ± 0.0117 | 0.8901 ± 0.0283 | 0.8787 ± 0.0214 | 0.798 ± 0.0429 | 0.8933 ± 0.0216 | 0.8111 ± 0.068 | 0.9493 ± 0.0094 | 0.9187 ± 0.0466 |
| | | **XGB** | 0.9261 ± 0.0076 | 0.8849 ± 0.0462 | 0.865 ± 0.014 | 0.786 ± 0.0841 | 0.8902 ± 0.0159 | 0.7954 ± 0.1071 | 0.9391 ± 0.0056 | 0.9173 ± 0.0493 |
| | | **MLP** | 0.8973 ± 0.0067 | 0.8754 ± 0.0442 | 0.8164 ± 0.01 | 0.7769 ± 0.0771 | 0.8582 ± 0.0118 | 0.8112 ± 0.094 | 0.9114 ± 0.0103 | 0.8988 ± 0.0499 |
| | | **SVC** | 0.8924 ± 0.0102 | 0.866 ± 0.0526 | 0.8032 ± 0.0189 | 0.7539 ± 0.0968 | 0.8259 ± 0.0226 | 0.7718 ± 0.1181 | 0.9165 ± 0.0076 | 0.9002 ± 0.055 |
| | | **LR** | 0.8548 ± 0.0069 | 0.8524 ± 0.054 | 0.7514 ± 0.0092 | 0.7507 ± 0.0785 | 0.8246 ± 0.0112 | 0.8229 ± 0.0849 | 0.8657 ± 0.0105 | 0.8631 ± 0.0673 |
| | | **KNN** | 0.9298 ± 0.0489 | 0.8618 ± 0.0334 | 0.8717 ± 0.0894 | 0.7469 ± 0.049 | 0.8876 ± 0.0784 | 0.7637 ± 0.0925 | 0.9452 ± 0.0384 | 0.8974 ± 0.0584 |
| | | **DT** | 0.8961 ± 0.008 | 0.8482 ± 0.0436 | 0.8039 ± 0.0163 | 0.7062 ± 0.0837 | 0.8018 ± 0.0376 | 0.6897 ± 0.1074 | 0.9303 ± 0.0146 | 0.9059 ± 0.0466 |
| B&C | 7 | **XGB** | 0.9474 ± 0.0107 | 0.8974 ± 0.0373 | 0.9013 ± 0.0196 | 0.8067 ± 0.0675 | 0.902 ± 0.0168 | 0.804 ± 0.1021 | 0.9639 ± 0.0089 | 0.9316 ± 0.0511 |
| | | **KNN** | 0.9257 ± 0.0394 | 0.8838 ± 0.039 | 0.8641 ± 0.0721 | 0.7921 ± 0.0593 | 0.8836 ± 0.0633 | 0.8185 ± 0.0598 | 0.9409 ± 0.0315 | 0.9073 ± 0.057 |
| | | **MLP** | 0.9089 ± 0.0075 | 0.8796 ± 0.0491 | 0.8334 ± 0.0136 | 0.7803 ± 0.0858 | 0.8569 ± 0.022 | 0.7992 ± 0.1039 | 0.9277 ± 0.0091 | 0.9087 ± 0.0575 |
| | | **RF** | 0.9517 ± 0.0227 | 0.8796 ± 0.0452 | 0.9092 ± 0.0423 | 0.7707 ± 0.0782 | 0.9046 ± 0.0346 | 0.7602 ± 0.1109 | 0.9688 ± 0.0189 | 0.923 ± 0.0575 |
| | | **SVC** | 0.9074 ± 0.0097 | 0.8775 ± 0.0429 | 0.8257 ± 0.0184 | 0.7687 ± 0.0817 | 0.8246 ± 0.0214 | 0.7675 ± 0.1124 | 0.9374 ± 0.0081 | 0.9173 ± 0.0546 |
| | | **LR** | 0.8542 ± 0.0066 | 0.8524 ± 0.0594 | 0.7446 ± 0.0107 | 0.7431 ± 0.0939 | 0.7988 ± 0.0131 | 0.7951 ± 0.1041 | 0.8743 ± 0.0074 | 0.8731 ± 0.0636 |
| | | **DT** | 0.908 ± 0.0208 | 0.8513 ± 0.052 | 0.8205 ± 0.0412 | 0.6987 ± 0.1083 | 0.7927 ± 0.0495 | 0.654 ± 0.121 | 0.9498 ± 0.0123 | 0.9229 ± 0.0377 |

**Continue on next page**

| Set | Number of features | Model | Accuracy ± sd | | F1 Score ± sd | | Sensitivity ± sd | | Specificity ± sd | |
|---|---|---|---|---|---|---|---|---|---|---|
| | | | Validation set | Test set | Validation set | Test set | Validation set | Test set | Validation set | Test set |
| IRONS core | 5 | RF | 0.7748 ± 0.0091 | 0.6911 ± 0.0429 | 0.4214 ± 0.0513 | 0.2016 ± 0.0606 | 0.3115 ± 0.0541 | 0.1457 ± 0.046 | 0.9426 ± 0.012 | 0.8888 ± 0.0536 |
| | | KNN | 0.7788 ± 0.0179 | 0.665 ± 0.0491 | 0.4667 ± 0.0368 | 0.1937 ± 0.0701 | 0.3645 ± 0.0378 | 0.1495 ± 0.0516 | 0.929 ± 0.0241 | 0.8517 ± 0.0553 |
| | | SVC | 0.7977 ± 0.0033 | 0.6775 ± 0.0527 | 0.4921 ± 0.0231 | 0.1931 ± 0.0935 | 0.3697 ± 0.032 | 0.1466 ± 0.079 | 0.9528 ± 0.0106 | 0.8702 ± 0.0621 |
| | | DT | 0.7639 ± 0.0111 | 0.6911 ± 0.0391 | 0.3587 ± 0.0635 | 0.1758 ± 0.0881 | 0.2516 ± 0.0567 | 0.1266 ± 0.0708 | 0.9496 ± 0.0102 | 0.8959 ± 0.0471 |
| | | MLP | 0.7179 ± 0.0029 | 0.7172 ± 0.0247 | 0.1353 ± 0.0143 | 0.1435 ± 0.0736 | 0.0831 ± 0.0099 | 0.0902 ± 0.0481 | 0.9479 ± 0.0046 | 0.9443 ± 0.029 |
| | | LR | 0.7359 ± 0.0024 | 0.7309 ± 0.0111 | 0.0377 ± 0.025 | 0.0222 ± 0.0358 | 0.0197 ± 0.0135 | 0.0118 ± 0.0191 | 0.9954 ± 0.0029 | 0.9914 ± 0.0138 |
| | | XGB | 0.7408 ± 0.0033 | 0.7256 ± 0.0084 | 0.0701 ± 0.0354 | 0.0 ± 0.0 | 0.0372 ± 0.0194 | 0.0 ± 0.0 | 0.9957 ± 0.003 | 0.9886 ± 0.0113 |
| A&B&C | 6 | XGB | 0.9275 ± 0.0094 | 0.8891 ± 0.0397 | 0.8677 ± 0.0168 | 0.7939 ± 0.0738 | 0.8937 ± 0.0157 | 0.8034 ± 0.0937 | 0.9398 ± 0.008 | 0.9202 ± 0.0426 |
| | | RF | 0.9425 ± 0.0179 | 0.8807 ± 0.0417 | 0.8946 ± 0.0315 | 0.7793 ± 0.0734 | 0.9121 ± 0.0184 | 0.7877 ± 0.0871 | 0.9536 ± 0.019 | 0.9145 ± 0.0507 |
| | | MLP | 0.9031 ± 0.0066 | 0.8764 ± 0.0493 | 0.8264 ± 0.0116 | 0.778 ± 0.0862 | 0.8675 ± 0.0178 | 0.8112 ± 0.1131 | 0.916 ± 0.0081 | 0.9002 ± 0.0586 |
| | | KNN | 0.9173 ± 0.0441 | 0.8712 ± 0.0393 | 0.849 ± 0.0805 | 0.7676 ± 0.0638 | 0.8687 ± 0.0706 | 0.7951 ± 0.0896 | 0.9349 ± 0.0347 | 0.8988 ± 0.0556 |
| | | SVC | 0.8884 ± 0.0082 | 0.8691 ± 0.0521 | 0.7959 ± 0.0142 | 0.7573 ± 0.0964 | 0.818 ± 0.0173 | 0.7678 ± 0.1181 | 0.9139 ± 0.0087 | 0.9059 ± 0.0571 |
| | | DT | 0.907 ± 0.0176 | 0.8619 ± 0.0507 | 0.8288 ± 0.0313 | 0.7428 ± 0.0956 | 0.8447 ± 0.0303 | 0.7528 ± 0.1241 | 0.9296 ± 0.0186 | 0.9016 ± 0.0552 |
| | | LR | 0.8555 ± 0.0062 | 0.8534 ± 0.0573 | 0.7449 ± 0.0097 | 0.7424 ± 0.0921 | 0.7931 ± 0.0134 | 0.7872 ± 0.0997 | 0.8781 ± 0.0082 | 0.8773 ± 0.0599 |
| ALL | 122 | XGB | 0.9661 ± 0.0111 | 0.8848 ± 0.0431 | 0.9356 ± 0.021 | 0.7818 ± 0.0809 | 0.9239 ± 0.0186 | 0.7798 ± 0.1157 | 0.9815 ± 0.009 | 0.923 ± 0.0522 |
| | | RF | 0.962 ± 0.0119 | 0.8649 ± 0.0486 | 0.9269 ± 0.023 | 0.7265 ± 0.0954 | 0.9073 ± 0.0258 | 0.6729 ± 0.0941 | 0.9818 ± 0.0077 | 0.9344 ± 0.0475 |
| | | LR | 0.8734 ± 0.0105 | 0.8408 ± 0.0557 | 0.7736 ± 0.0145 | 0.7211 ± 0.0711 | 0.8123 ± 0.0134 | 0.76 ± 0.0807 | 0.8955 ± 0.0151 | 0.8701 ± 0.0784 |
| | | MLP | 0.9805 ± 0.0155 | 0.8273 ± 0.0464 | 0.9611 ± 0.0317 | 0.6894 ± 0.0731 | 0.9356 ± 0.0662 | 0.7211 ± 0.114 | 0.9967 ± 0.0063 | 0.8659 ± 0.0697 |
| | | SVC | 0.8806 ± 0.0122 | 0.8211 ± 0.0585 | 0.7551 ± 0.0273 | 0.642 ± 0.0968 | 0.6929 ± 0.034 | 0.5986 ± 0.1056 | 0.9486 ± 0.0051 | 0.9015 ± 0.0721 |
| | | DT | 0.883 ± 0.0207 | 0.823 ± 0.0376 | 0.767 ± 0.0446 | 0.627 ± 0.0965 | 0.7279 ± 0.0671 | 0.5743 ± 0.1368 | 0.9391 ± 0.0199 | 0.913 ± 0.0477 |
| | | KNN | 0.9555 ± 0.0576 | 0.8156 ± 0.0298 | 0.9046 ± 0.1233 | 0.5786 ± 0.0671 | 0.8675 ± 0.1713 | 0.476 ± 0.0562 | 0.9873 ± 0.0165 | 0.9387 ± 0.027 |

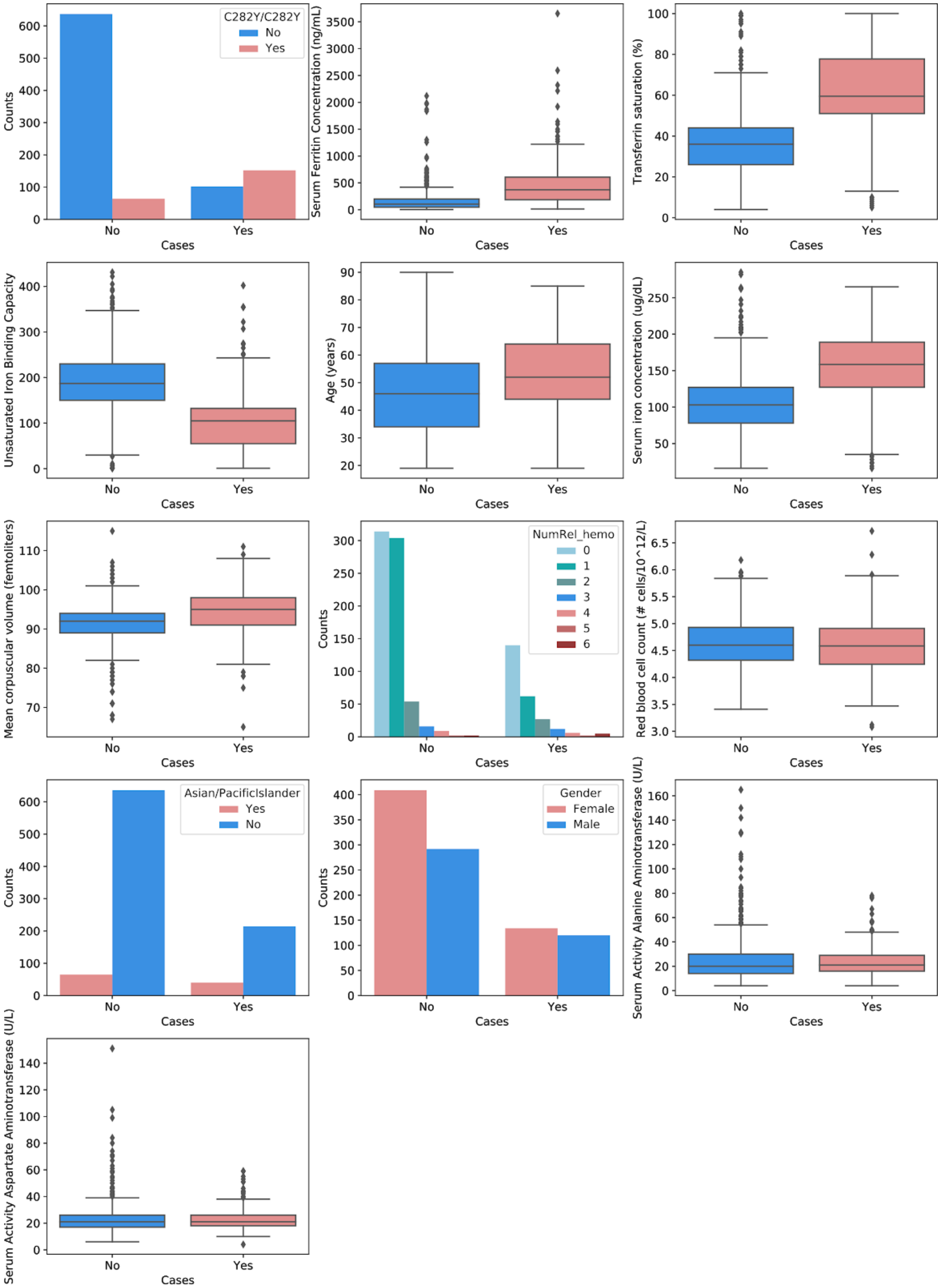


**Supplementary figure 1.** Distribution of the most relevant risk factors among healthy individuals and HH cases (Set B). Counts represent the number of individuals that fulfil the explored conditions.
